# Calcium Homeostasis Is Modified in Skeletal Muscle Fibers of Small Ankyrin1 Knockout Mice

**DOI:** 10.3390/ijms20133361

**Published:** 2019-07-09

**Authors:** Enrico Pierantozzi, Péter Szentesi, Dána Al-Gaadi, Tamás Oláh, Beatrix Dienes, Mónika Sztretye, Daniela Rossi, Vincenzo Sorrentino, László Csernoch

**Affiliations:** 1 Department of Molecular and Developmental Medicine, Molecular Medicine Section, University of Siena, 53100 Siena, Italy; 2 Department of Physiology, Medical Faculty, University of Debrecen, H-4002 Debrecen, Hungary; 3 Doctoral School of Molecular Medicine, University of Debrecen, H-4002 Debrecen, Hungary

**Keywords:** sarcoplasmic reticulum, sAnk1, calcium release, spark

## Abstract

Small Ankyrins (sAnk1) are muscle-specific isoforms generated by the *Ank1* gene that participate in the organization of the sarcoplasmic reticulum (SR) of striated muscles. Accordingly, the volume of SR tubules localized around the myofibrils is strongly reduced in skeletal muscle fibers of 4- and 10-month-old sAnk1 knockout (KO) mice, while additional structural alterations only develop with aging. To verify whether the lack of sAnk1 also alters intracellular Ca^2+^ handling, cytosolic Ca^2+^ levels were analyzed in stimulated skeletal muscle fibers from 4- and 10-month-old sAnk1 KO mice. The SR Ca^2+^ content was reduced in sAnk1 KO mice regardless of age. The amplitude of the Ca^2+^ transients induced by depolarizing pulses was decreased in myofibers of sAnk1 KO with respect to wild type (WT) fibers, while their voltage dependence was not affected. Furthermore, analysis of spontaneous Ca^2+^ release events (sparks) on saponin-permeabilized muscle fibers indicated that the frequency of sparks was significantly lower in fibers from 4-month-old KO mice compared to WT. Furthermore, both the amplitude and spatial spread of sparks were significantly smaller in muscle fibers from both 4- and 10-month-old KO mice compared to WT. These data suggest that the absence of sAnk1 results in an impairment of SR Ca^2+^ release, likely as a consequence of a decreased Ca^2+^ store due to the reduction of the SR volume in sAnk1 KO muscle fibers.

## 1. Introduction

The sarcoplasmic reticulum (SR) of skeletal muscle fibers is a convoluted organelle consisting of a network of membrane-limited tubules where Ca^2+^ is stored. Two distinct SR regions can be described: the longitudinal SR (l-SR) and the junctional SR (j-SR). The l-SR surrounds each myofibril throughout its entire length and represents the major site for Ca^2+^ uptake from the cytosol due to the presence of the sarco/endoplasmic reticulum Ca^2+^-ATPase (SERCA) pumps. The j-SR is formed by the so-called terminal cisternae that are connected to the l-SR tubules. In skeletal muscle, two terminal cisternae are positioned on opposite sides of a transverse (T)-tubule, an in-folding of the sarcolemma, to form the triad. The membrane of the terminal cisternae facing the T-tubule accommodates the ryanodine receptor (RyR1) Ca^2+^ release channels, and additional SR proteins, including triadin, junctin, and calsequestrin, which, together with RyR1 channels, participate in the mechanisms of excitation–contraction (e–c) coupling. This mechanism transduces depolarization-induced activation of the dihydropyridine receptor (DHPR), the voltage sensor on T-tubules, into a release of Ca^2+^ from the SR to allow muscle contraction [1]. Alterations of this mechanism are associated with different skeletal muscle diseases in humans [2,3,4,5], including Central and Multi Mini Core Disease, and centronuclear myopathies associated with mutations in RyR and myotubularin, respectively [6,7,8].

The correct positioning of the SR around myofibrils and the connection with T-tubules are essential for the efficient regulation of muscle contraction [9]. Ankyrins are a family of protein adaptors involved in the organization of specialized membrane domains. All three ankyrin genes (*Ank1*, *Ank2* and *Ank3*) encode, through multiple splicing events, a large number of ankyrin isoforms that share a common general structure [10]. Interestingly, in striated muscles, all three ankyrin genes code for muscle-specific isoforms [11,12,13,14]. In particular, in addition to the high molecular weight (220–130 kDa) that ankyrin isoforms present in most tissues, an internal promoter in the *Ank1* gene drives the expression of small muscle-specific isoforms of 20–25 kDa, collectively known as sAnk1 isoforms (sAnk1.5, sAnk1.6, sAnk1.7 and sAnk1.9). These sAnk1 isoforms lack most of the canonical ankyrin domains and contain a unique hydrophobic domain at their NH_2_-terminal, which localizes them in the SR [15,16,17,18]. Among these short muscle isoforms, sAnk1.5 is the most abundantly expressed, while the other isoforms are expressed at lower levels [19]. sAnk1.5 contains, within its cytoplasmic tail, a specific sequence that allows direct binding to obscurin [20,21], a sarcomeric protein required for the maintenance of M-band stability in skeletal muscle [22,23,24,25]. The interaction between sAnk1.5 and obscurin is crucial to maintain the SR in close proximity to the myofibrils [26]. Knockdown of sAnk1 isoforms in primary cultures of rat *flexor digitorum brevis* (FDB) resulted in a loss of anastomosis within tubules of the l-SR, while the organization of the j-SR was only barely affected [27]. In agreement with this observation, the more recent characterization of the sAnk1 knockout (KO) mouse, an animal model where sAnk1 expression is completely abolished, showed that the lack of sAnk1 results in a significant reduction of the SR volume, although RyRs and SERCA protein levels and localization did not differ in fibers from both fast and slow muscles of sAnk1 KO and WT mice [28]. Notably, almost identical alterations in SR morphology were also observed in skeletal muscle fibers from obscurin KO mice, confirming the relevance of the interaction between sAnk1 and obscurin in preserving SR architecture [29]. Further analysis revealed that, with age, fibers from sAnk1 KO mice developed structural damage of contractile elements, large areas of contracture and tubular aggregates containing SR proteins such as calsequestrin, SERCA1 and triadin, all involved in Ca^2+^ handling. These structural alterations were accompanied by an impairment of electrophysiological properties and force generation in sAnk1 KO muscles [28]. This evidence is consistent with a previous work reporting that cultured fibers where sAnk1 had been knocked down displayed a reduced Ca^2+^ release from the SR [27], indicating that structural alterations generated by the absence or reduced expression of sAnk1 may result in impaired Ca^2+^ handling. However, whether the deletion of sAnk1 also affects Ca^2+^ homeostasis in skeletal muscle fibers with respect to the phenotypes observed in young and old KO mice is far less known. Here, we report results from the analysis of Ca^2+^ fluxes in FDB fibers of sAnk1 KO mice, showing that fibers from KO mice have altered Ca^2+^ transients, and less frequent and smaller spontaneous Ca^2+^ release events compared to WT mice.

## 2. Results

### 2.1. Decreased SR Ca^2+^ Content in Skeletal Muscle Fibers from sAnk1 KO Mice

**Figure 1:**
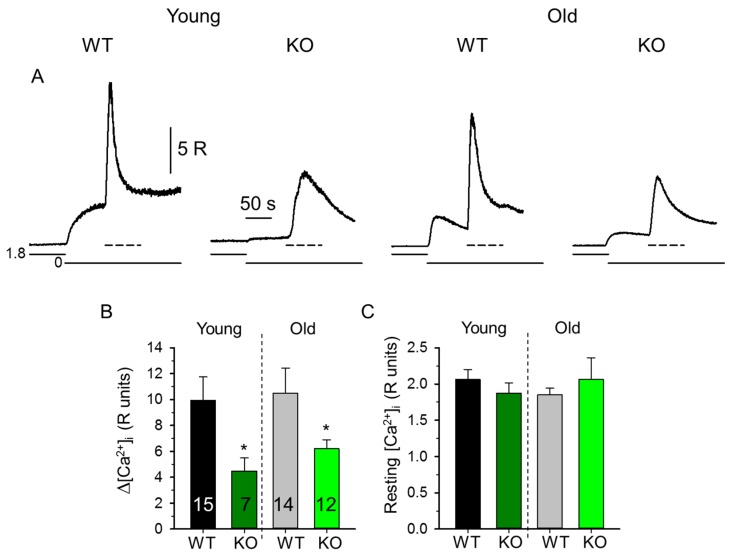
Decreased Ca^2+^ transients in *flexor digitorum brevis* (FDB) fibers from small ankyrin1 (sAnk1) knockout (KO) mice. (**A**) Representative depletion cocktail evoked Ca^2+^ transients measured on fibers isolated from the FDB muscles of 4- and 10-month-old wild type (WT) and sAnk1 KO mice. Pooled data for resting [Ca^2+^]_i_ (**C**), and maximal increase in [Ca^2+^]_i_ (**B**). The protocol of the solution exchange in panel A was: 0–70 s normal Tyrode’s solution (1.8, solid line), 70–300 s Ca^2+^-free Tyrode’s with 5 mM EGTA (0, solid line), 150–220 s releasing cocktail (200 µM 4-chloro-M-cresol and 10 µM cyclopiazonic acid) in Ca^2+^-free Tyrode’s (dashed line). Numbers in columns show the number of fibers averaged. The number of animals in groups varied between 3 and 4. * denotes significant difference from WT at *p* < 0.05.

The effects of the deletion of sAnk1 on Ca^2+^ homeostasis were examined in single FDB fibers isolated from 4- and 10-month-old mice. At first, we measured the resting [Ca^2+^]_i_ in unstimulated Fura-2 loaded FDB fibers from sAnk1 KO and WT mice. These measurements indicated that the resting Ca^2+^ levels were not significantly different in both 4- and 10-month-old FDB fibers from sAnk1 KO mice compared to WT mice (Figure 1C). In Figure 1A, representative Ca^2+^ transients following treatment with a releasing cocktail in a calcium-free extracellular solution are reported. The removal of Ca^2+^ from the extracellular solution occasionally depolarized the fibers, resulting in a slight increase in [Ca^2+^]_i_. The application of the releasing cocktail to muscle fibers from WT mice resulted in a large Ca^2+^ transient that decreased even in the continued presence of the cocktail, as a consequence of store emptying (Figure 1A). On the contrary, the average change in the amplitude of Ca^2+^ transients (Δ[Ca^2+^]_i_) was significantly smaller in fibers from 4- and 10-month-old KO mice with respect to age-matched control mice (Figure 1A,B), suggesting that the total SR Ca^2+^ content in sAnk1 KO mice is lower than that in WT mice.

**Figure 2:**
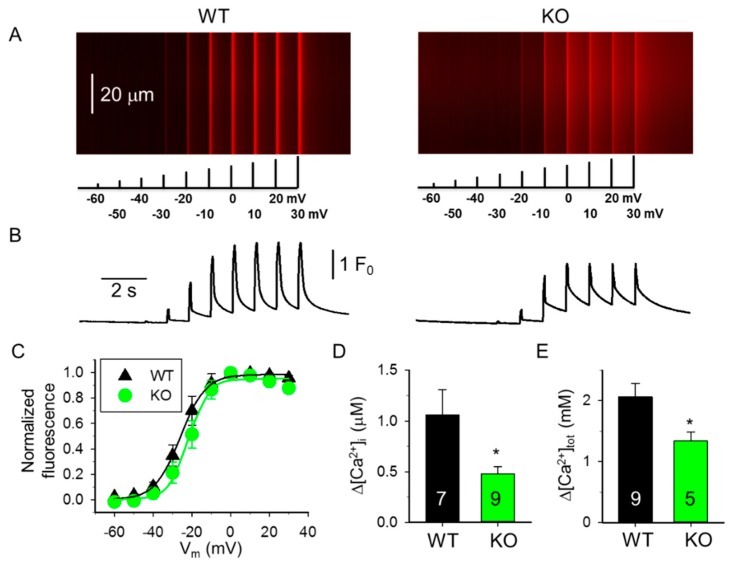
Membrane potential dependence of changes in [Ca^2+^]_i_ in fibers from 4-month-old WT and sAnk1 KO mice. (**A**) Line-scan images of changes in [Ca^2+^]_i_ elicited under whole-cell voltage-clamp by 100 ms long progressively increasing membrane depolarizations ranging between −60 mV and +30 mV, with 10 mV increments every 500 ms in WT and KO fibers. Each cell was held at −80 mV and perfused with 10 mM EGTA. The scale bar in the left panel corresponds to both line-scans. (**B**) Temporal profile of the changes in [Ca^2+^]_i_ calculated from the corresponding images in panel A by averaging 50 lines in the spatial domain normalized to average resting *F*_0_(*x*). (**C**) Voltage dependence of the changes in [Ca^2+^]_i_. Values obtained for individual fibers were first fitted by a Boltzmann function (Equation (1)) and then normalized to the obtained maximum for a given fiber, and finally averaged over the fibers. Continuous lines represent the best fit of the Boltzmann function to the average values with parameters of k = 8.95 and 6.17 mV, and V_50_ = −21.60 and −20.91 mV, for WT (*n* = 10) and KO (*n* = 9), respectively. (**D**,**E**) Pooled data for Δ[Ca^2+^]_i_ (**D**) and the total amount of Ca^2+^ released (**E**) at +30 mV depolarization from four WT and five KO mice. To assess the peak change in [Ca^2+^]_i_ (**D**) and the amount of Ca^2+^ released (**E**), 100 ms and 1 s long single pulses were used, respectively. Numbers in columns show the number of fibers averaged. * denotes significant difference from WT at *p* < 0.05.

**Figure 3:**
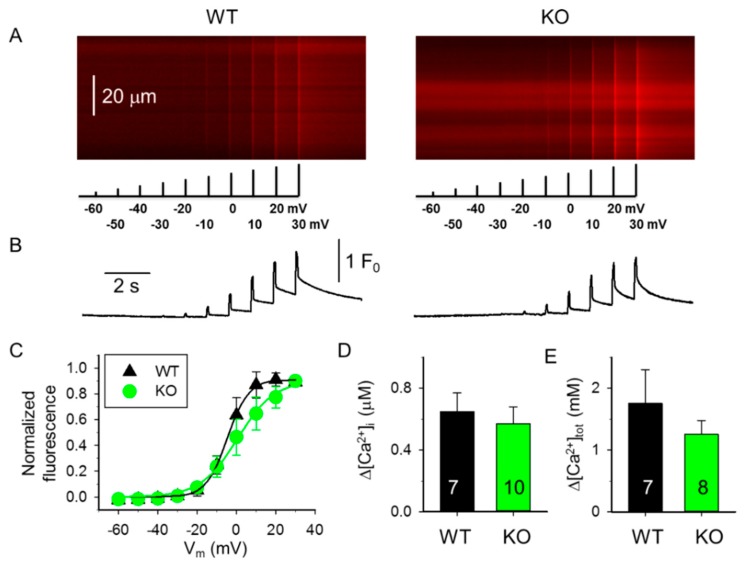
Membrane potential dependence of changes in [Ca^2+^]_i_ in 10-month-old WT and sAnk1 KO mice. (**A**) Line-scan images of changes in [Ca^2+^]_i_ elicited by the same depolarization protocol as in Figure 2. The scale bar in the left panel corresponds to both line-scans. (**B**) Temporal profile of the changes in [Ca^2+^]_i_ calculated from the corresponding images in panel A. (**C**) Voltage dependence of the changes in [Ca^2+^]_i_. Values were obtained as described for Figure 2C. Continuous lines represent the best fit of the Boltzmann function to the average values with parameters of k = 8.09 and 14.67 mV, and V_50_ = −5.94 and −7.86 mV, for WT (*n* = 9) and KO (*n* = 9), respectively. (**D**,**E**) Pooled data for Δ[Ca^2+^]_i_ (**D**) and amount of Ca^2+^ released (**E**) at +30 mV depolarization for three WT and five KO mice. To assess the peak change in [Ca^2+^]_i_ (**D**) and the amount of Ca^2+^ released (**E**), 100 ms and 1 s long single pulses were used, respectively. Numbers in columns show the number of fibers averaged.

We next induced changes in [Ca^2+^]_i_ under whole-cell voltage-clamp by progressively increasing membrane depolarization between −60 mV and +30 mV (Figure 2 and Figure 3). As shown in Figure 2A,B,D, the average change in the amplitude of Ca^2+^ transients in 4-month-old mice was significantly smaller in fibers from sAnk1 KO mice than from WT mice. When similar experiments were performed with 10-month-old mice (Figure 3A), a small difference between fibers from sAnk1 KO mice and WT was still observed, but it did not reach statistical significance (Figure 3B,D). Similarly, when the total amount of Ca^2+^ released during the depolarizing pulse at +30 mV was calculated, a significant difference between sANK1 KO mice and WT mice was observed in 4-month-old mice, but not in 10-month-old mice (Figure 2E and Figure 3E).

The voltage dependence of the transients was only slightly affected by sAnk1 deletion in both 4-month-old mice (Figure 2C) and 10-month-old mice (Figure 3C), although it was less steep in the older mice. Interestingly, the midpoint voltage of the Boltzmann curves was shifted by almost 20 mV to the right, i.e., to more positive voltages, in both 10-month-old mice groups (Table 1).

To evaluate whether the reduced Ca^2+^ transients observed following treatment with the releasing cocktail or induced by membrane depolarization may have resulted from a decreased Ca^2+^ transport by SERCA pumps, the maximal transport rate (V_max_) was calculated in fibers from WT and sAnk1 KO mice. This analysis revealed that the V_max_ progressively reduced in old mice compared to young animals, in both WT and sAnk1 KO mice (*p* = 0.0057 and *p* < 0.0001, respectively). In addition, in old sAnk1 KO mice the V_max_ was significantly lower compared to WT (1.68 ± 0.31 and 3.13 ± 0.49 mM s^−1^, *n* = 9 and 8, respectively; *p* = 0.028). This suggests that the remarkable but not statistically significant reduction in Ca^2+^ transients observed with age may be partially explained by a lower SERCA pump activity. However, the difference in Ca^2+^ transients observed between WT and sAnk1 KO muscle fibers at 4 months of age cannot be ascribed to SERCA pumps, since no difference could be observed between the two genotypes at this age (4.72 ± 0.08 and 4.79 ± 0.07 mM s^−1^, respectively; *p* = 0.54, *n* = 9 in both groups).

### 2.2. Spontaneous Ca^2+^ Release Events (CREs) Are Reduced in sAnk1 KO Mice

**Figure 4:**
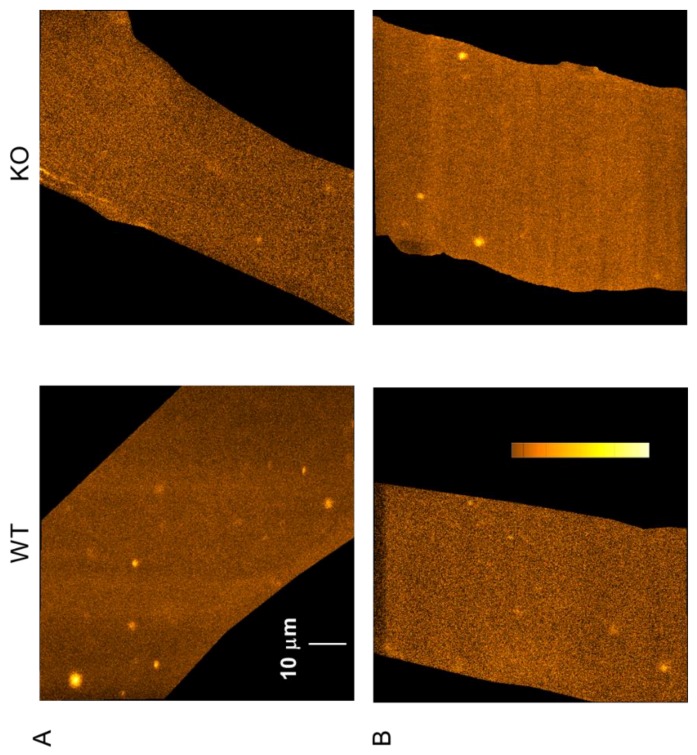
Spontaneous Ca^2+^ release events in saponin-permeabilized FDB fibers. Representative composite XY images from 4-month-old (**A**) and 10-month-old (**B**) WT (left panels) and sAnk1 KO (right panels) mice. Changes in [Ca^2+^]_i_ were visualized by loading the fibers with fluo-3. Each image is the composite of 120 images recorded with a frequency of 16 Hz. The scale bar in the top left image corresponds to all images. The color bar in the bottom left image corresponds to all images. Lighter colors represent a higher change in [Ca^2+^]_i_.

The measurement of spontaneous Ca^2+^ release events in saponin-permeabilized muscle fibers offers the possibility to study the functional properties of RyR1 in its native environment, independently from the control of the DHPR [30,31]. We used this method to test whether the lack of sAnk1 would affect the spontaneous Ca^2+^ release activity from the SR. Spontaneous CREs were measured using confocal XY imaging in fluo-3 loaded fibers isolated from 4- and 10-month-old KO and WT mice. Events shown in Figure 4 represent the cumulated CRE activity recorded during a series of 120 consecutive images, covering a period of approximately 20 s.

**Figure 5:**
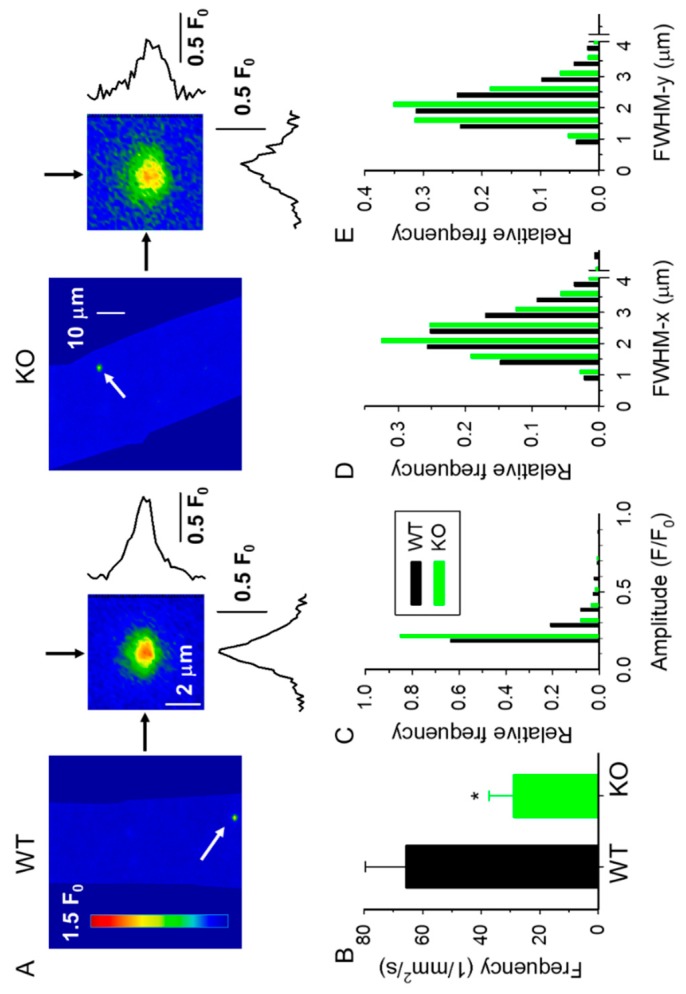
Parameters of Ca^2+^ release events in 4-month-old WT and sAnk1 KO mice. (**A**) A single XY image of normalized fluorescence (F/F_0_). The images were obtained by selecting one image from a series (as presented in Figure 4) and normalizing to baseline fluorescence (F_0_) after removing the background (determined outside of the fiber). Enlarged portions (black arrows) present the Ca^2+^ release events (CREs) marked in the original image together with their spatial profiles parallel (x) or perpendicular (y) to the longitudinal axis of the fiber. (**B**–**E**) Pooled data for CRE frequency (**B**), amplitude (**C**), and full width at half maximum (FWHM) in x (**D**) and in y (**E**) directions for WT (number of events, *n* = 1084) and KO (*n* = 1231) fibers.

**Figure 6:**
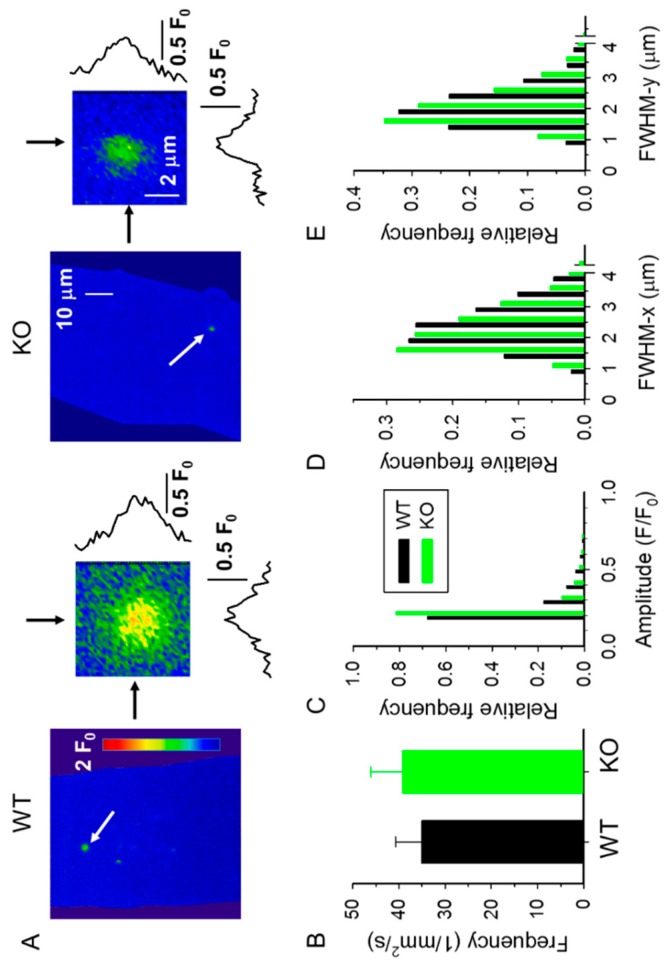
Parameters of Ca^2+^ release events in 10-month-old WT and sAnk1 KO mice. (**A**) A single XY image of normalized fluorescence (F/F_0_). The images were obtained as described for Figure 5A. (**B**–**E**) Pooled data for CREs frequency (**B**), amplitude (**C**), and FWHM in x (**D**) and in y (**E**) directions for WT (number of events, *n* = 1063) and KO (*n* = 1646) fibers.

Single XY images and CREs from 4- and 10-month-old mice are shown on an enlarged scale in Figure 5A and Figure 6A, respectively. A significant reduction in the frequency of CREs was detected in fibers from 4-month-old sAnk1 KO mice as compared to age-matched WT mice (Figure 5B, Table 2). The frequency of CREs declines in fibers from 10-month-old WT mice, and no further difference was observed between KO and WT mice (Figure 6B, Table 2).

We next analyzed the mean CRE amplitude (F/F_0_) and observed a significant decrease in 4- and 10-month-old KO mice compared to their age-matched WT counterparts (Table 2). The analysis of the distribution of the amplitude of CREs revealed a higher frequency of CREs with a small amplitude in KO fibers compared to age-matched WT fibers (Figure 5C and Figure 6C). When the spatial widths of CREs, namely full widths at half maximum (FWHMs) perpendicular to, and parallel to, the Z-lines (i.e., FWHM-x and FWHM-y) were analyzed, a significant reduction of these parameters was observed in fibers from both 4- and 10-month-old KO mice (Table 2), with an increased number of small CREs in fibers from KO mice (Figure 5D,E, and Figure 6D,E). These alterations were clearly reflected in the calculated amount of Ca^2+^ released, estimated as the Signal Mass (SM) during the CREs. As reported in Table 2, the absence of sAnk1 was accompanied by a marked reduction in SM, independently of the age of the mice. These data indicate that there is either a reduction in the Ca^2+^ available for immediate release in the SR or an impaired opening of the Ca^2+^ release channels, or both.

## 3. Discussion

In skeletal muscle fibers, the SR has acquired specific morphological adaptations to efficiently sustain Ca^2+^ release for muscle contraction [1,32,33]. In particular, sAnk1, localized on the SR membrane, binds to the sarcomeric giant protein obscurin. This protein–protein interaction is necessary for the accurate positioning of the SR tubules, where Ca^2+^ is stored, around the myofibrils, in order to regulate muscle contraction. We previously showed that a lack of sAnk1 results in SR volume reduction in 4-month-old mice, with the appearance of contractures, tubular aggregates and myofibrillar disarray in aging sAnk1 KO mice. All these events are compatible with imbalanced intracellular Ca^2+^ levels [25], which, with age, may lead to the muscle damage observed in old animals.

In this study, we investigated the effect of deletion of sAnk1 on Ca^2+^ homeostasis in skeletal muscle fibers from 4- and 10-month-old sAnk1 KO mice. Our results indicate that total SR Ca^2+^ content was reduced in 4- and 10-month-old sAnk1 KO mice compared to age-matched WT mice, as revealed by the use of a releasing cocktail. Ca^2+^ transients induced in voltage-clamped fibers were reduced also in muscle from 4- but not from 10-month-old sAnk1 KO mice, compared to those from age-matched WT mice. Indeed, the amplitude of Ca^2+^ transients in 10-month-old WT mice was reduced compared to that observed in 4-month-old WT mice. This is in agreement with previously reported data indicating that aging leads to a physiological decline of Ca^2+^ release from the SR [34]. On the contrary, voltage-induced Ca^2+^ transients in fibers from 4-month-old sAnk1 KO mice were similar to those obtained from fibers isolated from 10-month-old sAnk1 KO mice. This is not surprising since the amplitude of Ca^2+^ transients in 4-month-old sAnk1 KO mice was reduced compared to that of age-matched WT mice. The voltage-induced Ca^2+^ transients measured in 10-month-old sAnk1 mice were comparable to those observed in 10-month-old WT mice. These results are consistent with the finding that sAnk1 KO mice show a reduction in the SR volume already in young animals, which may explain the decrease in the amplitude of Ca^2+^ transients. Interestingly, the voltage dependence of SR Ca^2+^ release was not affected in sAnk1 KO fibers, suggesting that the altered Ca^2+^ release observed in sAnk1 KO fibers might reflect a reduction of the Ca^2+^ store, likely due to the decrease in l-SR volume observed in sAnk1 KO mice [28], rather than to a defect in the DHPR-induced RYR1 activation.

An additional piece of evidence suggesting a reduction of the Ca^2+^ store in skeletal muscle fibers from sAnk1 KO mice was obtained by measuring CRE parameters. CREs represent elementary events of Ca^2+^ release through RyR1 channels, activated by cytosolic and/or luminal Ca^2+^ [35]. Results obtained with saponin-permeabilized fibers, which represent an experimental model where luminal Ca^2+^ content is the most relevant driving force for sparks to occur, clearly showed that the amplitude, spatial spread and frequency of CREs were all reduced in muscle fibers from KO mice compared to WT. In addition, signal mass values calculated from spark parameters, which in turn represent an indirect assessment of the Ca^2+^ released [36], were significantly lower in sAnk1 KO fibers, further supporting a decreased Ca^2+^ content in the SR of fibers lacking sAnk1. Given that RyRs have a calcium sensor on their luminal side which, if occupied, stimulates the opening of the channel [37], it is possible to envisage that the reduced SR content might be responsible for the reduced spark frequency.

Furthermore, the indirect measurement of SERCA pump activity in WT and sAnk1 KO mice did not reveal a significant difference between the two genotypes in young animals. Only in 10-month-old sAnk1 KO mice, the V_max_ of SERCA pump resulted to be significantly decreased. Considering that the reduction in SR volume is comparable between young and old sAnk1 KO mice, the marked decrease in SERCA pump activity observed in 10-month-old sAnk1 KO mice may not be caused by the SR morphology nor by a loss of interaction between sAnk1.5 and SERCA and/or sarcolipin [38,39], but it may rather result from the structural alterations observed in skeletal muscles of old sAnk1 KO mice [28], although yet unidentified additional cues cannot actually be ruled out. Alternatively, compensatory events that may be active in young sAnk1 KO mice may no longer suffice in 10-month-old sAnk1 KO mice.

In conclusion, our results show that the deletion of sAnk1.5 results in an impairment of Ca^2+^ homeostasis in skeletal muscle fibers of young sAnk1 KO mice, compared to age-matched controls. The observed difference in Ca^2+^ homeostasis becomes less evident when, with age, Ca^2+^ release efficiency also decreases in the skeletal muscle of old WT mice [34]. This suggests that in young sAnk1 KO mice Ca^2+^ handling is already compromised at levels comparable to those observed in old WT mice. On the other hand, with age, KO mice develop structural alterations, like tubular aggregates and contractures that are not present in WT mice. Future work is required to better address the mechanisms affected by the deletion of sAnK1, impaired Ca^2+^ homeostasis and the development of structural alterations.

## 4. Materials and Methods

### 4.1. Animal Care

The generation of sAnk1 KO mice has been described in Giacomello et al. [28]. The mouse colony was maintained by intercrossing heterozygous mice, and the genotype of the progeny was determined by polymerase chain reaction as previously described [28]. The experiments were carried out on sAnk1 KO and littermate wild type mice weighing 20–30 g. The animals were kept under standard laboratory conditions (12 h light/dark cycle, room temperature 22–25 °C) with free access to tap water and pelleted mouse chow in their plastic cages, which have mesh covers. Animal experiments followed the guidelines of the European Community (86/609/EEC) and the experimental protocol (31/2012/DE MAB) was approved by the institutional Animal Care Committee of the University of Debrecen. 

Animals were anaesthetized and sacrificed following a protocol (22/2011/DE MÁB, 2012) approved by the Animal Care Committee of the University of Debrecen. After pentobarbital anesthesia (27 mg/kg) and cervical dislocation, the *m. flexor digitorum brevis* from the hind limbs were dissected.

### 4.2. Isolation of Single Skeletal Muscle Fibers

All Ca^2+^ concentration measurements were carried out on skeletal muscle fibers from the FDB muscles of control (WT) and sAnk1 deficient (KO) male mice of both 4 and 10 months of age. Ca^2+^ free Tyrode’s solution (in mM: 137 NaCl, 5.4 KCl, 0.5 MgCl_2_, 1.8 CaCl_2_, 11.8 Hepes-NaOH, 1 g/L glucose, pH 7.4) was used during the dissection of the muscle. Single muscle fibers from FDB were enzymatically dissociated in a minimal essential medium containing 0.2% Type I collagenase (Sigma, St Louis, MO, USA) at 37 °C for 35 min [37]. To obtain single intact fibers, FDB muscles were triturated gently in normal Tyrode’s solution (1.8 mM CaCl_2_). Fibers were then placed in culture dishes and stored at 4 °C in refrigerator until use.

### 4.3. Whole Cell Intracellular Ca^2+^ Concentration Measurements

Changes in intracellular Ca^2+^ concentration ([Ca^2+^]_i_) in different extracellular solutions were measured using a Fura-2 fluorescent calcium indicator as described previously [40]. Briefly, enzymatically isolated single FDB fibers were mounted on a laminin-coated glass cover slip and loaded with 5 µM Fura-2 acetoxymethyl ester (AM) for 1 h. Fibers were then kept in normal Tyrode’s solution for half an hour at room temperature. Measurements were carried out on an inverted fluorescence microscope (Nikon Diapoth, Nikon, Tokyo, Japan). A microcomputer-controlled dual-wavelength monochromator alternated the excitation wavelength with 50 Hz between 340 and 380 nm (Deltascan, Photon Technology International, New Brunswick, NJ, USA), whereas a photomultiplier measured the emission signal at 510 nm with a 10 Hz acquisition rate at room temperature. To measure SR Ca^2+^ content, isolated FDB fibers were permanently washed with Tyrode’s solution using a background perfusion system, then a calcium-free Tyrode’s solution with 5 mM EGTA was applied through a local perfusion system, which was positioned in close proximity to the selected fiber. Intracellular Ca^2+^ stores were emptied using a depleting cocktail (200 µM 4-chloro-M-cresol and 10 µM cyclopiazonic acid) in calcium-free Tyrode’s resulting in a massive Ca^2+^-release from the SR via RyRs. All solutions contained 50 µM BTS to minimize fiber movement artefacts during calcium release. [Ca^2+^]_i_ was assessed as the ratio of measured fluorescence intensities (F_340_/F_380_). Resting [Ca^2+^]_i_ was calculated from averaging the ratio in normal Tyrode’s. The SR Ca^2+^ content was calculated as the difference between resting [Ca^2+^]_i_ and the maximum of the ratio measured during the application of the releasing cocktail.

### 4.4. Measurement of Ca^2+^ Release Events (CREs) in Permeabilized Muscle Fibers

Isolated FDB muscle fibers were permeabilized with saponin and loaded with 0.1 mM fluo-3 using experimental solutions and procedures previously described [41]. In brief, fibers were bathed in a relaxing solution (in mM: 6 MgCl_2_, 5 Na_2_-ATP, 125 K-glutamate, 10 HEPES, 1 EGTA, 0.13 CaCl_2_, 10 glucose, 10 Na-phosphocreatine, pH 7.2) containing 0.002% saponin for 2–3 min. Permeabilization of the surface membrane by the detergent was monitored by imaging how the fluorescence of fluo-3 increases in the muscle fiber. This solution was then replaced by a recording solution (in mM: 6 MgCl_2_, 5 Na_2_-ATP, 95 K_2_SO_4_, 10 HEPES, 1 EGTA, 0.13 CaCl_2_, 10 glucose, 10 Na-phosphocreatine, pH 7.2). Images were recorded with a 40× oil immersion objective (NA = 1.3) on a Zeiss LSM 5 LIVE laser scanning confocal microscope (Zeiss, Oberkochen, Germany). The 488 nm line of an argon laser excited fluo-3 in the fibers, and the emitted fluorescent light was collected above 505 nm wavelength. Fifteen minutes following the application of the recording solution, eight consecutive series of hundred and twenty 512 × 512 (x, y) images captured every 67 ms were collected in each tested fiber. CRE detection and analysis were performed using modified methods and algorithms previously described in [42]. In brief, the region of the background and the contour of the fiber were defined on the first image of each series. Then, the averaged background fluorescence calculated from the selected area was subtracted from each pixel of all images in one series. To detect the Z-lines in the fiber, the frequency spectrum in each line of all images was calculated using fast Fourier transform (FFT). Then, inverse FFT of the frequency components corresponding to sarcomeres was used to remove the Z-lines from the images. The stationary wavelet method with soft threshold filtering was used to detect Ca^2+^ release events on the fibers. Finally, the following spark parameters were calculated: the amplitude and two full widths at half maximum (FWHMs), perpendicular to (FWHM-x) and parallel with (FWHM-y) the Z-lines. 

To assess the amount of Ca^2+^ released during a CRE, the Signal Mass (SM) was calculated as described in [43]. To estimate FWHM in the *z* direction (i.e., the optical axis), the average of the two FWHMs in the focal plane (*x* and *y* directions) was used.

### 4.5. Voltage Clamp and Ca^2+^ Transient Analysis

The experimental design was similar to the one described in [44]. Briefly, isolated fibers were voltage-clamped (Axoclamp 2B, Axon Instruments, Foster City, CA, USA) and imaged using a confocal microscope (Zeiss LSM 5 Live) in external solution (in mM: 140 TEA-CH_3_SO_3_, 1 CaCl_2_, 3.5 MgCl_2_, 10 Hepes, 1 4-Aminopyridine, 0.5 CdCl_2_, 0.3 LaCl_3_, 0.001 TTX, and 0.05 BTS, pH 7.2, osmolality 320 mOsm). Fibers were dialyzed with rhod-2 containing internal solution (in mM: 110 N-methylglucamine, 110 L-glutamic acid, 10 EGTA, 10 Tris, 10 glucose, 5 Na ATP, 5 phosphocreatine Tris, 0.1 rhod-2, 3.56 CaCl_2_, and 7.4 mM MgCl_2_, pH 7.2, osmolality 320 mOsm). All experiments were conducted at 20–22 °C and the resting holding potential was kept at −80 mV. The pipette resistance varied in the range of 1–2 MΩ. Analog compensation was used to correct the linear capacitive currents.

Ca^2+^ transients were analyzed by fitting a removal model that calculates release flux. This takes into account the evolution of [Ca^2+^]_i_(t) in a single compartment including quantitatively specified processes of removal, such as the maximal rate of the SERCA pump (V_max_) [45]. The experiments were performed in the presence of calcium buffer (10 mM EGTA), thus the endogenous buffers were considered almost negligible in the removal process. The Boltzmann function was used to describe the voltage dependence of the activation of Ca^2+^ release:

Δ[Ca^2+^]_i,max_/(1 + exp(−(V_m_ − V_50_)/k) (1)

where V_50_ is the midpoint voltage and the reciprocal of k is the limiting logarithmic of the slope. Line-scan images were analyzed by a custom-made program using the following parameters: K_d rhod-2_ = 18 μM and k_ON_ = 0.7 10^8^ M^−1^s^−1^. From the release flux (the flux exiting through the release channels), the net flux leaving the SR can be derived by subtraction of the pump removal flux. The integral of the net flux provides the SR content releasable by depolarization or otherwise termed the amount of Ca^2+^ released (Δ[Ca^2+^]_tot_).

### 4.6. Chemicals and Statistical Analysis

Chemicals, unless otherwise stated, were purchased from Sigma (St. Louis, MO, USA) and were of analytical grade.

Pooled data were expressed as mean ± standard error of the mean (SEM). The differences between control and sAnk1 KO mice with different ages were assessed using two-way analysis of variance (ANOVA) and all pairwise multiple comparison procedures (Student-Newman-Keuls Method) with Prism (GraphPad Software, San Diego, CA, USA). An f-test was used to test the significance and a *p* value of less than 0.05 was considered statistically significant.

## Figures and Tables

**Table 1:** Parameters describing the voltage dependence of the depolarizing pulse-evoked Ca^2+^transients in WT and sAnk1 KO fibers.

Boltzmann Parameters †	WT 4-Month-Old (4 Mice) (9 Fibers)	KO 4-Month-Old (5 Mice) (9 Fibers)	WT 10-Month-Old (4 Mice) (9 Fibers)	KO 10-Month-Old (4 Mice) (9 Fibers)
max	1.91 ± 0.27	1.11 ± 0.19 *	1.61 ± 0.34	1.39 ± 0.32
k (mV)	7.25 ± 1.09	5.57 ± 0.85	5.88 ± 0.92	8.67 ± 1.16 ^#^
V_50_ (mV)	−21.84 ± 4.96	−20.68 ± 2.97	−4.76 ± 4.44 ^#^	−3.62 ± 6.17 ^#^

† Parameters were obtained by fitting the voltage dependence of the individual fibers using Equation (1). * indicates significant difference from WT at *p* < 0.05. ^#^ indicates significant difference from young mice from the same genetic background at *p* < 0.05.

**Table 2:** Parameters of CREs in WT and sAnk1 KO fibers.

	WT 4-Month-Old (3 Mice) (43 Fibers)	KO 4-Month-Old (9 Mice) (87 Fibers)	WT 10-Month-Old (3 Mice) (76 Fibers)	KO 10-Month-Old (4 Mice) (105 Fibers)
Number of sparks	1084	1231	1063	1646
Amplitude (F/F_0_)	0.216 ± 0.005	0.158 ± 0.003 ***	0.205 ± 0.006	0.171 ± 0.003 ***^,##^
FWHM-x (µm)	2.239 ± 0.023	2.048 ± 0.020 ***	2.312 ± 0.025 ^#^	1.972 ± 0.020 ***^,##^
FWHM-y (µm)	1.968 ± 0.020	1.784 ± 0.017 ***	1.991 ± 0.022	1.761 ± 0.017 ***
Signal mass (SM) (µm^3^)	3.195 ± 0.130	1.852 ± 0.090 ***	2.909 ± 0.124	1.996 ± 0.088 ***
Frequency (mm^−2^ s^−1^)	65.8 ± 13.8	28.8 ± 8.6 *	35.0 ± 5.7 ^#^	39.1 ± 6.9

* and *** indicate significant difference from WT at *p* < 0.05 and *p* < 0.001, respectively. ^#^ and ^##^ indicate significant difference from young mice from the same genetic background at *p* < 0.05 and *p* < 0.01, respectively.
